# Full-length 16S rDNA sequencing based on Oxford Nanopore Technologies revealed the association between gut-pharyngeal microbiota and tuberculosis in cynomolgus macaques

**DOI:** 10.1038/s41598-024-53880-w

**Published:** 2024-02-10

**Authors:** Vorthon Sawaswong, Prangwalai Chanchaem, Pavit Klomkliew, Suwatchareeporn Rotcheewaphan, Suthirote Meesawat, Taratorn Kemthong, Mutchamon Kaewparuehaschai, Kirana Noradechanon, Monya Ekatat, Reka Kanitpun, Prapaporn Srilohasin, Saradee Warit, Angkana Chaiprasert, Suchinda Malaivijitnond, Sunchai Payungporn

**Affiliations:** 1 Department of Biochemistry, Center of Excellence in Systems Microbiology, Faculty of Medicine, Chulalongkorn University, 1873 Rama IV Road, Patumwan, Bangkok, 10330 Thailand; 2 Department of Biochemistry, Faculty of Science, Mahidol University, Bangkok, 10400 Thailand; 3 Department of Microbiology, Faculty of Medicine, Chulalongkorn University, Bangkok, 10330 Thailand; 4 National Primate Research Center of Thailand, Chulalongkorn University, Saraburi, 18110 Thailand; 5 Department of Biology, Faculty of Science, Chulalongkorn University, Bangkok, 10330 Thailand; 6 Wildlife Conservation Office, Department of National Parks Wildlife and Plant Conservation, Bangkok, 10900 Thailand; 7 National Institute of Animal Health (NIAH), Bangkok, 10900 Thailand; 8 Office for Research, Faculty of Medicine Siriraj Hospital, Mahidol University, Bangkok, 10700 Thailand; 9 Department of Microbiology, Faculty of Medicine Siriraj Hospital, Mahidol University, Bangkok, 10700 Thailand; 10 Industrial Tuberculosis Team, Industrial Medical Molecular Biotechnology Research Group, National Center for Genetic Engineering and Biotechnology, National Science and Technology Development Agency, Pathum Thani, 12120 Thailand

**Keywords:** Tuberculosis, Cynomolgus macaque, Gut-pharyngeal microbiome, Full-length 16S sequencing, Microbiome, Zoology

## Abstract

Tuberculosis (TB) is an infectious disease caused by the *Mycobacterium tuberculosis* complex (*Mtb*c), which develops from asymptomatic latent TB to active stages. The microbiome was purposed as a potential factor affecting TB pathogenesis, but the study was limited. The present study explored the association between gut-pharyngeal microbiome and TB stages in cynomolgus macaques using the full-length 16S rDNA amplicon sequencing based on Oxford Nanopore Technologies. The total of 71 macaques was divided into TB (−) control, TB (+) latent and TB (+) active groups. The differential abundance analysis showed that *Haemophilus hemolyticus* was decreased, while *Prevotella species* were increased in the pharyngeal microbiome of TB (+) macaques. In addition, *Eubacterium coprostanoligenes* in the gut was enriched in TB (+) macaques. Alteration of these bacteria might affect immune regulation and TB severity, but details of mechanisms should be further explored and validated. In summary, microbiota may be associated with host immune regulation and affect TB progression. The findings suggested the potential mechanisms of host-microbes interaction, which may improve the understanding of the role of microbiota and help develop therapeutics for TB in the future.

## Introduction

Tuberculosis (TB) is an infectious disease caused by *Mycobacterium tuberculosis* complex (*Mtb*c)^1^. There are 10 million cases of TB newly developed annually and 1.2 million deaths worldwide^2^. The disease severity ranges from the asymptomatic (latent TB infection, LTBI) stage to active stage^3^. Most cases typically develop LTBI, while only 5–15% of infected patients have the progression to active TB, causing pulmonary infiltration or disseminated disease^4^. The *Mtb*c in the latent phase can persist within the granuloma surrounded by aggregated immune cells^1^. Previous studies found that factors such as human immunodeficiency virus (HIV) infection^5^ and tumor necrosis factor (TNF) may dysregulate the immune system, causing the failure to suppress *Mtb*c within granuloma, and the disease is reactivated to be active stage^6^. Nevertheless, other factors associated with TB progression have complicated and unclear mechanisms.

Recently, several studies suggested that the bacterial community or microbiome in humans could be one of the most critical factors involving TB progression^7,8^. It was found that dysbiosis of the microbiome in the gut could promote the progression and severity of the disease. For example, the change of microbiota caused by antibiotic consumption can increase the TB severity^9^. The infection of gut pathogens such as *Helicobacter* spp. also affected the susceptibility of TB challenge^10^. In addition, the gut microbiome could also communicate and regulate the respiratory (lung) immune via immune cells translocated through the lymphatic system and producing metabolites such as short-chain fatty acids (SCFAs)^8^. This regulation mechanism was called the gut-lung axis. The lung microbiome also regulates innate and acquired immune responses for TB. Increasing anaerobes producing SCFAs such as *Prevotella* and *Veillonella* in the lung could inhibit some protective cytokines, which may promote the progression of TB^11^. The microbiome in oropharyngeal cavity is also comprised of diverse microbes including bacteria in genera *Prevotella, Campylobacter, Veillonella, Streptococcus* and *Neisseria* which may have a protective role in respiratory tract infections (RTIs)^12^. On the other hand, the acute pathogen infection could disturb the pharyngeal microbiome homeostasis and lead to secondary infection as the previous analysis^13^. In the case of the recent COVID-19 pandemic, the confirmed cases of COVID-19 have opportunistic pathogens increased while butyrate-producing genera such as *Bifidobacterium, Fusobacterium,* and *Porphyromonas* decreased^14^. Another study showed that the relative abundances of the genera *Streptococcus, Veillonella, Prevotella,* were increased while *Haemophilus, Fusobacterium,* and *Gemella* spp. were decreased in COVID-19 compared to the healthy group^15^ suggesting the association of microbiome related to RTIs which could also be useful as clinical indicators. In addition, the pharyngeal microbiome changes by reduction of *Anaerococcus, Corynebacterium* and *Streptococcus* may influence the development of allergic rhinitis modulating immune responses^16^. This supports the idea that the oropharyngeal microbiome may be associated with immune responses and infectious diseases including TB. Therefore, the relationship between the gut-respiratory microbiome and *Mtb*c infection has become motivating in research. However, the studies of microbiomes and TB in humans could be complicated due to various confounding factors such as diets, habitat, and lifestyles^17^. Thus, animals that can be representative models for studying the microbiome and TB with controllable factors become a focus.

Cynomolgus macaque (*Macaca fascicularis*) is the most commonly encountered macaque species in Thailand^18^. They highly interacted with humans and were found to be naturally infected with *Mtb*c^19^. Cynomolgus macaques had high resistance to *Mtb*c^20,21^, and the infected macaques were generally in the LTBI stage which were similar to those found in humans^22^. Thus, the LTBI macaques could be the reservoir of *Mtb*c, which may spillover to humans. Recently, researchers from the NPRCT-CU and the Industrial Tuberculosis Team of the National Science and Technology Development Agency (NSTDA) reported that 36% of the macaque population (or 14 out of 39 animals) at Krabok-Koo Wildlife Breeding Center, Chachoengsao, Thailand were the *Mtb*c exposure^23^. Some macaques showed an active stage with severe symptoms, and some were at the LTBI stage. However, it is still unclear what factors might promote the progression from LTBI to the active stage in these *Mtb*c*-*infected macaques.

This study aimed to investigate the relationship between the gut-respiratory microbiome and TB stages in cynomolgus macaques using microbiome profiling based on high-throughput sequencing. Because the study of lung microbiome needs an invasive method for specimen collection, the pharyngeal microbiota representing the partial-upper respiratory microbiome became the focal point of this study. Exploring the pharyngeal microbiota could represent the lung (lower respiratory) microbiota since the oropharyngeal microbes are the primary source of lung microbiome^24^. In addition, pharyngeal microbiota would be better for investigating potential airborne pathogens. Therefore, the full-length 16S rDNA amplicon sequencing based on Oxford Nanopore Technologies was performed to explore the gut and pharyngeal microbiome diversity and abundance in macaques. Intensive computational analysis has been conducted to extract useful information and interpret microbiome sequencing data. The findings inferred the potential functions of the microbiome and host-microbe interactions, which may help understand *Mtb*c infection and TB stages in humans.

## Results

### Bacterial diversity in the gut and pharyngeal microbiota

**Figure 1:**
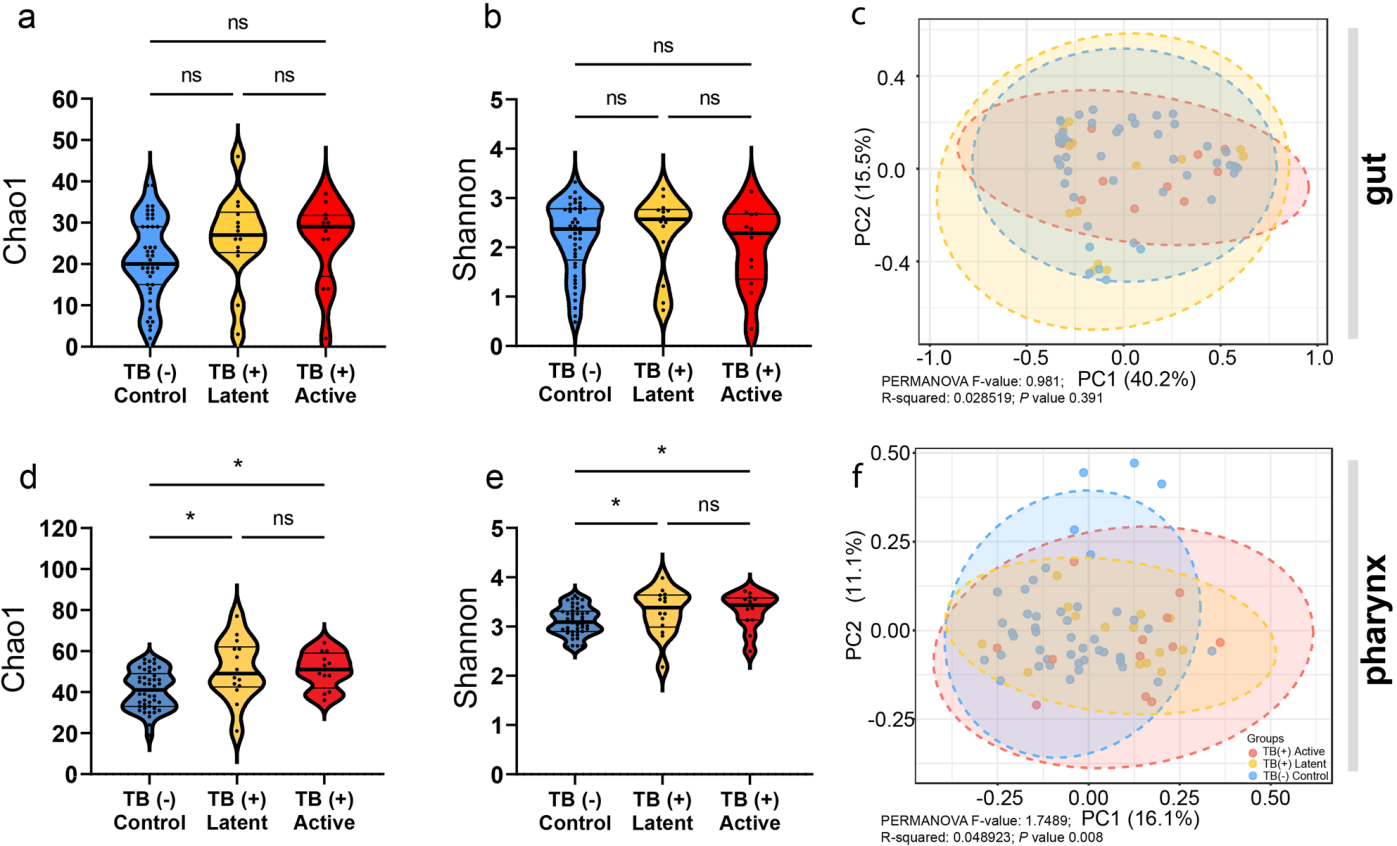
The diversity of gut and pharyngeal microbiota of macaques. The alpha diversity of macaque microbiota with different TB stages was evaluated by Chao1 and Shannon diversity indexes and tested with the Kruskal–Wallis test (*P* < 0.05). The alpha diversity of gut microbiota was shown in (**a**) Chao1 and (**b**) Shannon diversity, while those of pharyngeal microbiota were presented in (**d**) and (**e**), respectively. The beta diversity of macaque microbiota with different TB stages was evaluated by the Bray–Curtis dissimilarity index and statistically tested by the PERMANOVA test (*P* < 0.05). The beta diversities were represented by Principal coordinate analysis (PCoA) plots as shown in (**c**) gut and (**f**) pharyngeal microbiota.

The rarefaction analysis of full-length 16S reads from amplicon sequencing was carried out, which indicated that the number of reads in the current experiment was enough for sampling the taxa in gut and pharyngeal microbiota, as shown in Supplementary Fig. S1a,b. The alpha diversity was determined by Chao1 and Shannon diversity indexes. Chao1 and Shannon diversity were not significantly different for gut microbiota (Kruskal–Wallis test, *P* = 0.11 and *P* = 0.49, respectively) among the three groups, as shown in Fig. 1a,b. However, the bacterial richness of pharyngeal microbiota differed among groups (*P* = 2.6 × 10^−3^). The post hoc analysis indicated a significant increase in richness was observed in TB (+) latent and TB (+) active groups (Fig. 1d). Similarly, the Shannon diversity was also significantly increased (*P* = 0.03) in both TB (+) groups, as presented in Fig. 1e, however, no significant difference in alpha diversity of pharyngeal microbiota between TB (+) latent and TB (+) active was observed. The beta diversity of gut and pharyngeal microbiota was investigated based on the Bray–Curtis dissimilarity index. The results showed that the gut microbiota of macaques (Fig. 1c) was not different among groups (PERMANOVA test, *P* = 0.26), whereas the pharyngeal microbiota was significantly different (PERMANOVA test, *P* = 8.00 × 10^−3^) as shown in Fig. 1f. To identify further whether dispersion or community profile was different in pharyngeal microbiota, the analyses of PERMDISP and ANOSIM tests were carried out, respectively. The dispersion of microbiota was not different among groups (PERMDISP, *P* = 0.46). However, The ANOSIM test showed that microbiota composition significantly differed among macaques with different TB stages (ANOSIM, *P* = 1.40 × 10^−2^).

### Relative abundance of gut and pharyngeal microbiota based on 16S amplicon sequencing

**Figure 2:**
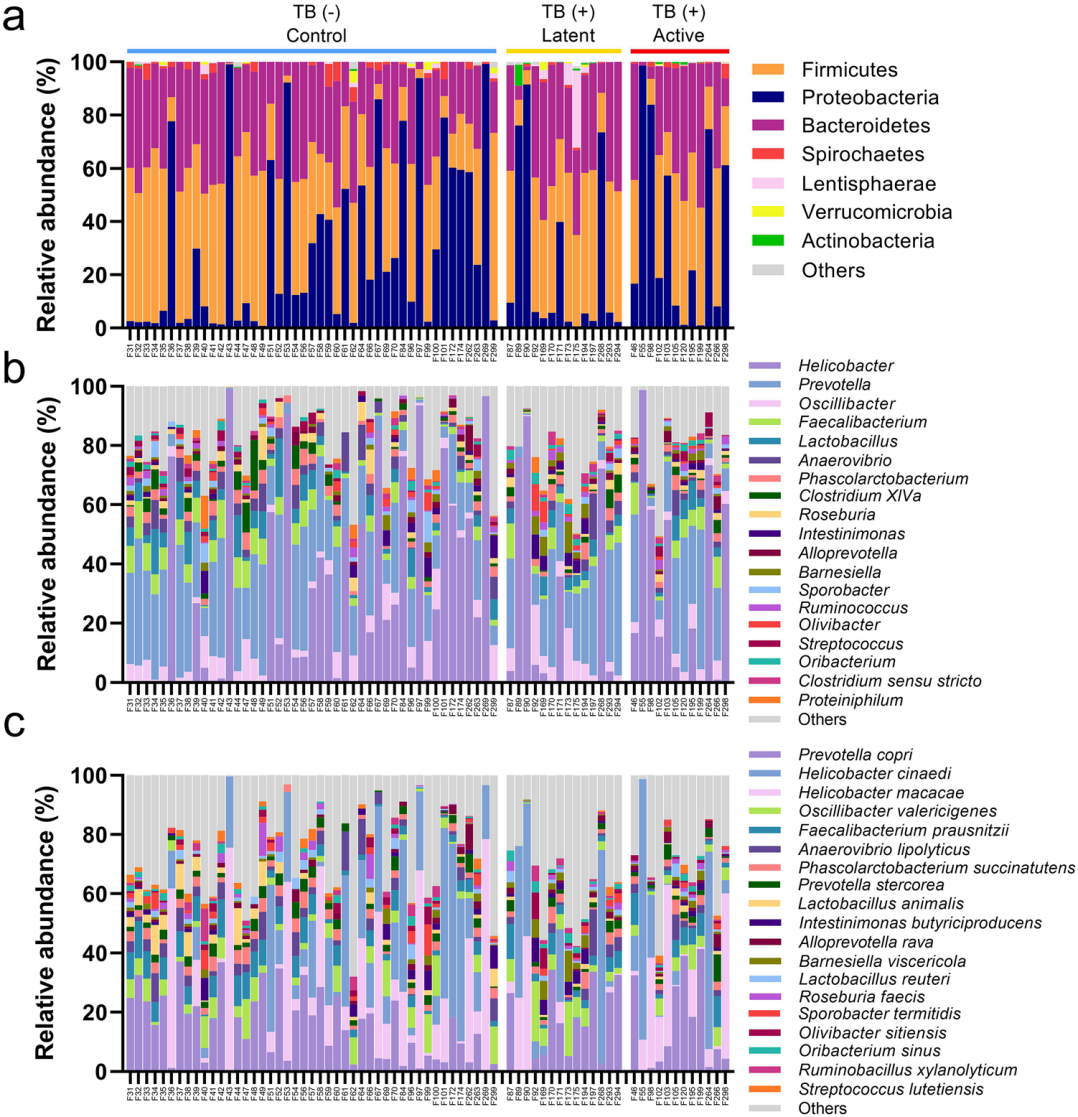
Relative abundance of gut microbiota in macaques with different TB stages. The relative abundances (%) of gut microbiota in macaques with different TB stages based on full-length 16S sequencing were shown as stacked bar plots based on 3 taxonomic ranks, including (**a**) phylum, (**b**) genus and (**c**) species.

**Figure 3:**
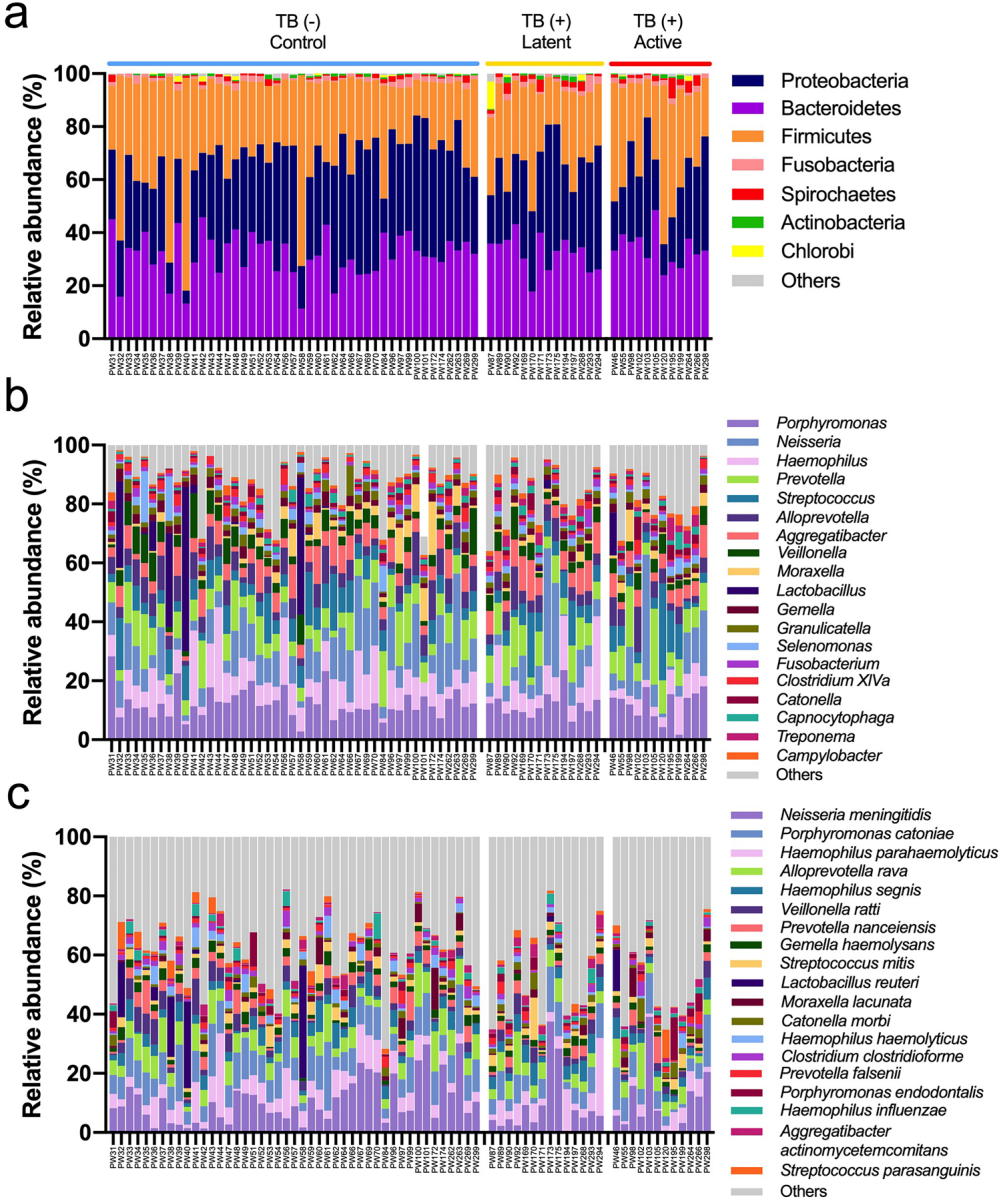
Relative abundance of pharyngeal microbiota in macaques with different TB stages. The relative abundances (%) of pharyngeal microbiota in macaques with different TB stages based on full-length 16S sequencing were shown as stacked bar plots based on 3 taxonomic ranks, including (**a**) phylum, (**b**) genus and (**c**) species.

The relative abundance of bacteria in the gut and pharyngeal microbiota was presented in Figs. 2 and 3, respectively. For gut microbiota, the phylum abundance indicated the high variation of microbiota profiles among individuals in Fig. 2a. Most macaques had two dominant phyla, Firmicutes and Bacteroidetes, which dominate the gut microbiota. The average abundance of Firmicutes and Bacteroidetes was 36.5 ± 19.0% and 29.7 ± 15.1%, respectively. Interestingly, some macaques had a high proportion of Proteobacteria (the abundance ranged from 0.5 to 99%). The abundance at the genus level indicated that *Helicobacter* was the main genus belonging to Proteobacteria found in gut microbiota which had 28.4 ± 33.0% of overall relative abundance Fig. 2b. The second most prevalent genus was *Prevotella* (21.7 ± 13.8%), whereas other genera had lower abundance (2 to 6%), including *Oscillibacter*, *Faecalibacterium*, *Lactobacillus*, *Anaerovibrio*, *Phascolarctobacterium*, and *Clostridium XlVa.* The abundance of species is presented in Fig. 2c. The top abundance species was *Prevotella copri,* contributing 15.5 ± 12.3% of total bacteria. The dominant *Helicobacters* found in these macaques can be divided into 2 different species: *Helicobacter cinaedi* (15.0 ± 21.0%) and *Helicobacter macacae* (12.6 ± 21.0%). Most *Oscillobacter* were classified as *Oscillibacter valericigenes* (4.8 ± 4.3%). Other high prevalent species had the average abundance lower than 5% such as *Faecalibacterium prausnitzii* (4.5 ± 3.6%), *Anaerovibrio lipolyticus* (3.0 ± 3.4%), *Phascolarctobacterium succinatutens* (2.3 ± 1.6%), *Prevotella stercorea* (2.3 ± 1.7%), *Lactobacillus animalis* (1.7 ± 2.7%)*,* and *Intestinimonas butyriciproducens* (1.4 ± 2.1%).

The phylum abundance of pharyngeal microbiota (Fig. 3a) showed more evenness of phyla composition than those in gut microbiota. This pharyngeal microbiota was mainly occupied by 3 major phyla, including Proteobacteria (33.1 ± 12.1%), Bacteroidetes (32.1 ± 7.8%), and Firmicutes (30.6 ± 13.2%). At the genus level (Fig. 3b), the pharyngeal microbiota was dominated by 2 genera, including *Porphyromonas* (12.2 ± 4.8%) and *Neisseria* (11.9 ± 9.2%). The less dominant taxa were *Haemophilus* (9.2 ± 6.26%), *Prevotella* (9.2 ± 4.76%), *Streptococcus* (7.1 ± 5.4%), *Alloprevotell*a (6.1 ± 3.4%), *Aggregatibacter* (6.1 ± 3.1%), and *Veillonella* (4.7 ± 3.1%). Figure 3c shows the top 20 species identified in the pharyngeal microbiome. The most abundant species was *Neisseria meningitidis,* contributing 10.4 ± 8.8% of the community. The second and third most abundant species were *Porphyromonas catoniae* (9.1 ± 4.5%) and *Haemophilus parahaemolyticus* (5.9 ± 5.0%). Other prevalent species were such as *Alloprevotella rava* (6.0 ± 3.4%), *Haemophilus segnis* (3.7 ± 2.2%), *Veillonella ratti* (3.0 ± 2.5%), *Prevotella nanceiensis* (2.5 ± 2.4%), *Gemella haemolysans* (2.2 ± 0.9%), and *Streptococcus mitis* (2.0 ± 2.1%).

### Cooccurrence network analysis of gut microbiota and pharyngeal microbiota

**Figure 4:**
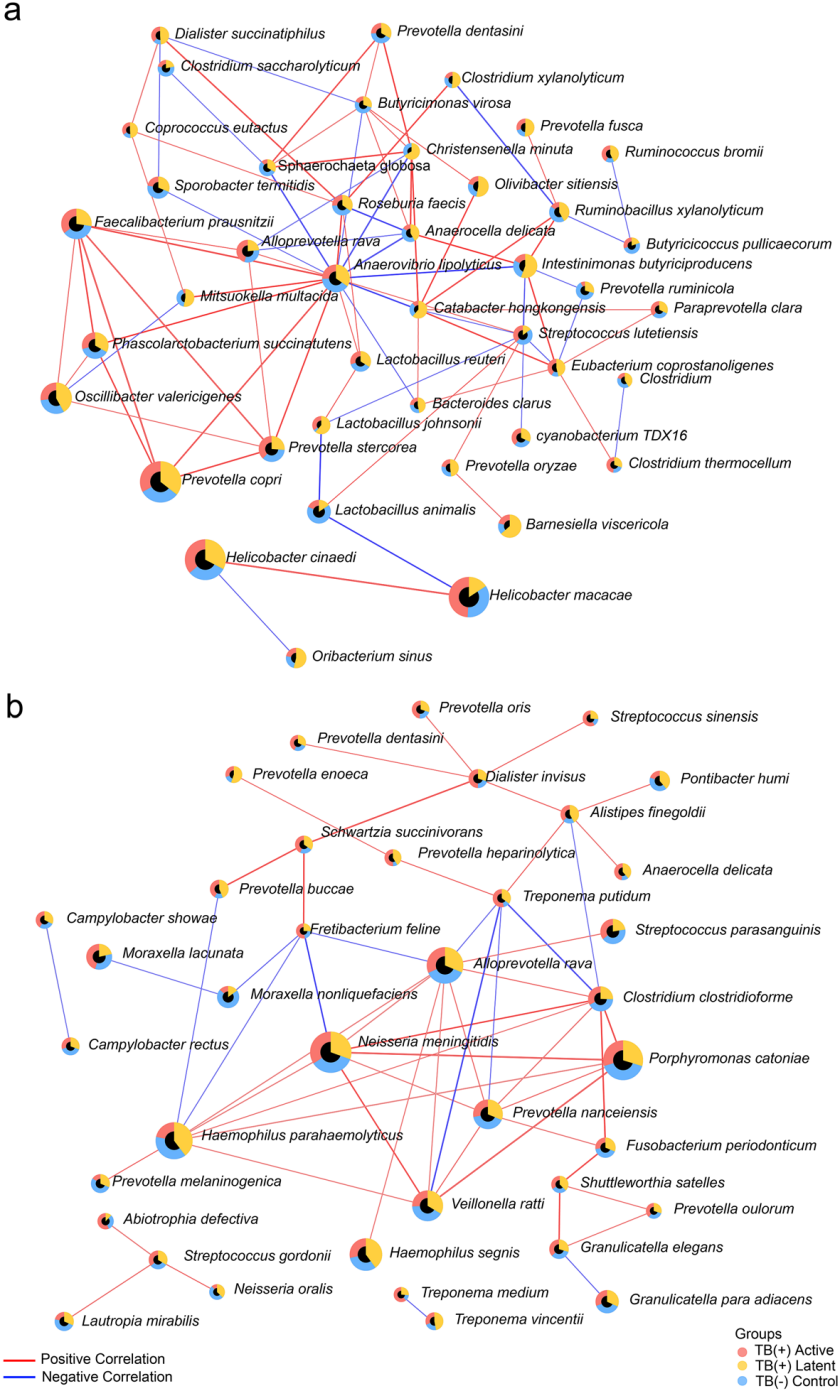
Correlation network analysis of gut and pharyngeal microbiota in cynomolgus macaques. The correlation network constructed based on the SPARCC method represents the relationship of bacterial species in the (**a**) gut and (**b**) pharyngeal microbiota of macaques (*r* > 0.3,* P* < 0.05). The colors of the edges represent correlation types: positive (red) and negative (blue) correlation. The nodes were designated as pie charts indicating the relative abundance of each bacterium in macaques with different TB stages: negative control (blue), latent (yellow) and active (red).

The occurrence network analysis was performed using the Sparse Correlations for Compositional Data (SparCC) method. Bacterial species in gut microbiota (Fig. 4a) demonstrated that the high-abundance bacteria, including *P. copri*, *O. valericigenes, F. prausnitzii*, *A. lipolyticus, P. succinatutens*, *P. stercorea*, *L. animalis,* and *A. rava* clustered together and showed a high correlation of abundance. The two abundant *Helicobacter* species (*H*. *cinaedi* and *H*. *macacae*) presented a strong correlation to each other but separated from the cluster of major abundant taxa described above. However, the analysis at the genus level showed that *Helicobacter* negatively correlated with *Lactobacillus,* as shown in Supplementary Fig. S2a. For the pharyngeal microbiome, the positive correlations of dominant species, including *N. meningitidis, H. parahaemolyticus, A. rava, P. catoniae,* and *P. nanceiensis* were observed in Fig. 4b. At the genus level, *Streptococcus*, *Neisseria*, and *Haemophilus* showed a solid correlation in the network presented in Supplementary Fig. S2b.

### Differential abundance analysis of microbiota in macaques with different TB stages

**Figure 5:**
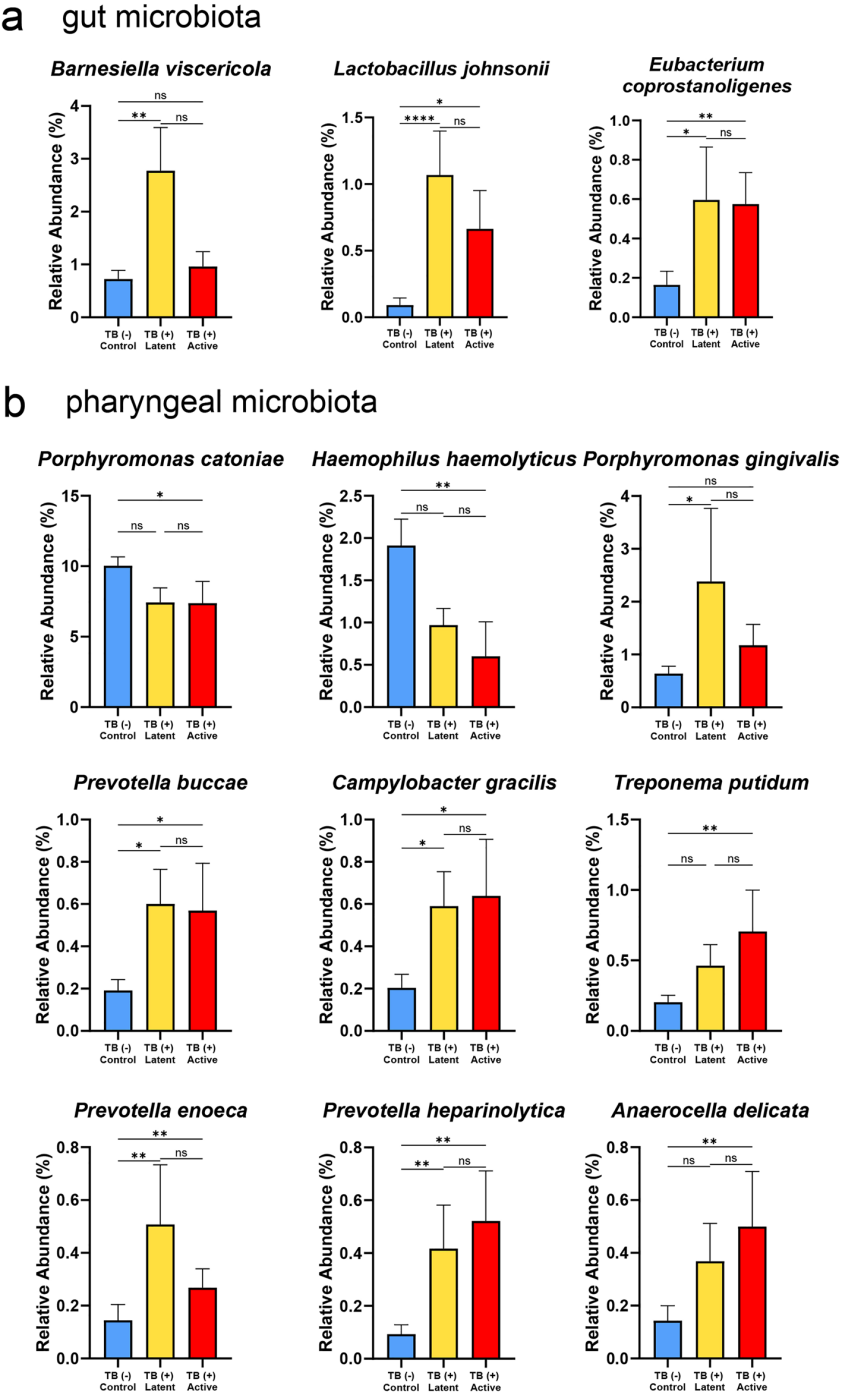
Differential abundance analysis of significant species in the gut and pharynx of macaques with different TB stages. The bar graphs showed the abundance of significant species from LEfSe analysis of (**a**) gut and (**b**) pharyngeal microbiota, which were statistically compared by the Kruskal–Wallis test (*P* < 0.05).

The LEfSe analysis was first carried out to identify the enriched taxa in macaque groups with different TB stages. For gut microbiota, the TB (−) group representing TB naive macaque had no significant enriched taxa (LDA score > 2, *P* < 0.05) in gut microbiota Supplementary Fig. S3. In contrast, few species significantly increased in the gut of TB (+) latent or TB (+) active groups. The Kruskal–Wallis test was parallelly performed to check the robustness of significant species (*P* < 0.05) and differences between groups showing the consistent significant species in Fig. 5a. It was found that *Barnesiella viscericola* significantly predominated in the gut of TB (+) latent macaques. *Lactobacillus johnsonii* and *Eubacterium coprostanoligenes* were greatly enriched in both TB (+) latent and active groups.

The pharyngeal microbiota had more significant enriched taxa from the LEfSe analysis Supplementary Fig. S4. At the genus level, several genera, such as *Bacteroides*, *Blautia*, *Desulfovibrio* and *Treponema* were enriched in the TB (+) active group, while the TB naive macaque had the enrichment of *Haemophilus*. The Kruskal–Wallis test presented the differentially enriched species among groups, as shown in Fig. 5b. *Porphyromonas catoniae* and *Haemophilus haemolyticus* were significantly enriched in the TB naive group. *Porphyromonas gingivalis* was enriched in only the TB (+) latent group. Some species, including *Treponema putidum* and *Anaerocella delicata,* were significantly dominant in TB (+) active macaques. The other species, including *Prevotella buccae, Campylobacter gracilis*, *Prevotella enoeca*, and *Prevotella haparinolytica,* increased considerably in both TB (+) latent and active groups.

It was generally known that gender factor may influence the accuracy as to describe the relationship between microbiome and TB. Therefore, we carried out the additional multivariate analysis based on the linear model (LM). The gender was used as the covariate to control the effect on the results comparing the relationship between microbiome and TB stages. The results showed concordant findings to the previous analysis (as shown in Supplementary Table 3–4) Additionally, the inferred absolute abundance was calculated and used for statistical analysis (carried out by ANCOM-BC^25^) to confirm the findings reported in this study. The *P* value and adjusted log fold-change were shown in Supplementary Table 5–6). The inferred absolute abundance of feathered taxa in Fig. 5 was still significant which ensures the association between these taxa and the stage of tuberculosis in long-tailed macaques.

### Functional inference of gut and pharyngeal microbiota based on the full-length 16S sequencing

**Figure 6:**
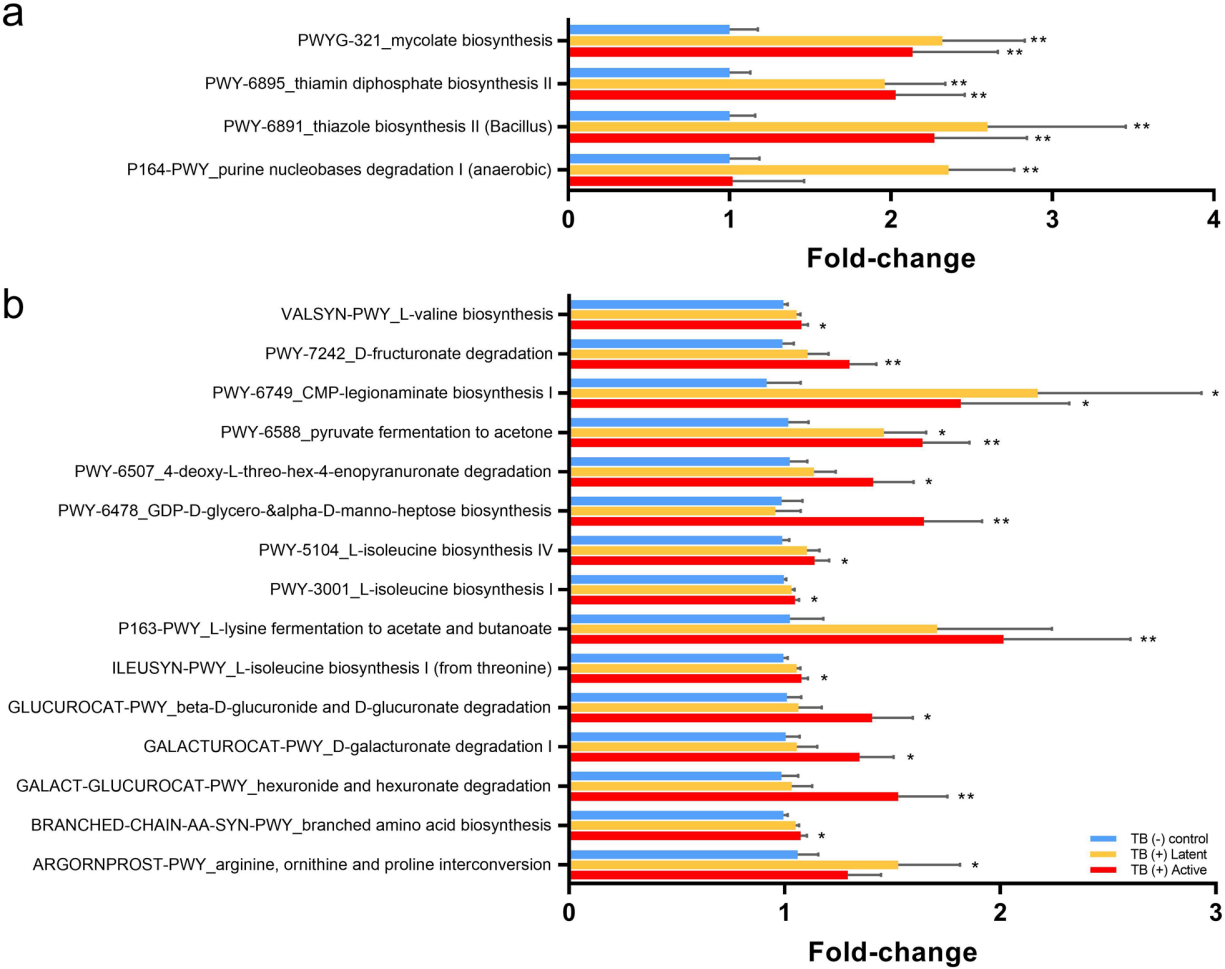
Functional inference analysis of microbiota in macaques with different stages of TB. The bar graphs present the fold-change of pathway abundance in each TB (+) group compared to the TB (−) control group based on PICRUSt2 analysis and MetaCyc pathway annotation. The pathway abundance was compared by *t*-test (*P* < 0.05). The significant pathways in gut microbiota were shown in (**a**), while those in pharyngeal microbiota were presented in (**b**).

The functional inference of microbiota was investigated based on PICRUSt2 software. For gut microbiota, only a few significant pathways (*t*-test, *P* < 0.05) were identified (Fig. 6a). It was found that the purine nucleobases degradation I (P164-PWY) was enriched in the TB (+) latent group. Other three pathways related to the biosynthesis of thiazole (PWY-6891), thiamin (PWY-6895), and mycolate (PWYG-321) were significantly abundant in both TB (+) latent and TB (+) active groups. The significant pathways in pharyngeal microbiota are shown in Fig. 6b. Several pathways related to the biosynthesis of amino acids were enriched in the TB (+) active group (such as PWY-5104, PWY-3001, VALSYN-PWY and ILEUSYN-PWY). The pathway of L-lysine fermentation to acetate and butanoate (P163-PWY) was also upregulated in the TB (+) active group. Similarly, the pathways associated with the degradation of galacturonate (GALACTUROCAT-PWY), glucuronate (GLUCUROCAT-PWY), hexuronate (GALACT-GLUCUROCAT-PWY), and fructuronate (PWY-7242) were abundant in the pharyngeal microbiota of TB (+) active macaques. In addition, the pyruvate fermentation to acetone pathway PWY-6588 and CMP-legionaminate biosynthesis I pathway (PWY-6749) enriched in both TB (+) latent and active groups.

## Discussion

This is the first study presenting the association of gut-(partial) upper respiratory microbiota and natural *Mtb*c infection in cynomolgus macaques. The full-length 16S sequencing based on Oxford Nanopore Technologies was utilized to explore the microbiota profiles. The alpha diversities of gut microbiota among the three TB stages were not different, whereas the diversity of pharyngeal microbiota was increased in TB (+) groups. The gut microbiota of these macaques may not be a significant factor influencing *Mtb*c infection and development. In contrast, the increasing microbial diversity in pharyngeal microbiota of TB (+) groups might be caused by the dysregulation of the immune response in the respiratory tract during TB development. The beta diversity showed that the gut microbiota profiles of the three macaque groups were not different, while the pharyngeal microbiota was shifted in the TB (+) groups. This suggested the strong relationship between pharyngeal microbiota and TB in these macaques. The relative abundance showed that the gut microbiota varied among samples. This gut microbiota may be affected by many factors, such as diets, environmental exposure, or the health status of macaques^26^. Since the macaques in the current study were reared in the same housing condition (more than 1 year), they might be normalized for the diets feeding and environmental exposure. Thus, the main possibly factor influencing their microbiota may be the health and immune status of an individual.

For the gut microbiota, the *Prevotella* spp. in macaque’s gut including *P. copri* and *P. stercorea* were also the common taxa in the human gut microbiome^27^. The less abundant species such as *P. succinatutens*^28^ and *F. prausnitzii*^29^ were also well-known and commonly found in the human gut. In contrast, other taxa were different from those in humans, but they were closely related to the same genus. The pharyngeal microbiome structure was very similar to humans when focused on the top abundant genera including *Prevotella, Veillonella, Streptococcus* and *Neisseria*. At species level, only some species such as *H. parahaemolyticus*^30^ and *H. haemolyticus*^31^ were suggested to be the common bacteria in the human pharynx. Although the species of bacteria were different, the members of the same genus could contribute similar functions in macaques and humans. Thus, the current finding may not directly represent the human microbiome, but it could infer the mechanisms and characteristics of microbiota related to TB.

Some macaques had a high abundance of *Helicobacter* spp., classified into 2 species, including *H. cinaedi* and *H. macacae*. It was reported that *H. cinaedi* could cause diarrhea after inoculation in the southern pig-tailed macaque (*M. nemestrina*)^32^ and was an opportunistic bacterium in humans^33^. In addition, *H. cinaedi* was isolated from the colon and liver of rhesus macaques (*M. mulatta*) with chronic colitis and hepatitis^33^. Similarly, *H. macacae* was isolated from rhesus and cynomolgus macaques with diarrhea from chronic idiopathic colitis^34,35^. Both *Helicobacter* spp. had a highly positive correlation in their relative abundance and a negative correlation with *Lactobacillus.* These bacteria may be increased after gut microbiota dysbiosis or alteration of the mucosal immune system. It has been previously reported that infection of *Helicobacter pylori* also affected the susceptibility of TB challenge in the mice model^10^. However, the current study showed that there was no relationship between *Helicobacter* spp. and TB in these macaques. This might be because the macaques were not directly challenged with *Mtb*c since we focused on naturally *Mtb*c*-*infected macaques. In addition, these macaques were reared in the gang cages which helped limit the transmission of *Mtb*c between different cages. This means that some macaques with high *Helicobacter,* may have not been exposed to *Mtb*c. Therefore, it is difficult to conclude the effect of *Helicobacter* on *Mtb*c susceptibility. Moreover, based on the current study and our previous report^36^, the *Helicobacter macacae and Helicobacter cinaedi* were found in healthy macaques even in the captive macaque in the primate center. Thus, it is possible that these *Helicobacter* species may not always cause diseases in healthy immunocompetent individuals. However, further study on these bacteria is needed to better explain their function and effect on macaque health.

The differential abundance analysis suggested that some gut bacteria, including *Lactobacillus johnsonii* and *Eubacterium coprostanoligenes,* were significantly increased in TB (+) latent and active groups. Nevertheless, this finding was different from the previous analysis of human microbiota related to TB. For example, Huang et al*.* reported that phylum Proteobacteria increased in active TB group while for LTBI individuals, genera *Prevotella* and *Roseburia* enriched^37^. However, the microbiota profiles related to TB in humans varied among studies^38^. This may be because the gut microbiota is associated with diets, and ethnicity^39^. Most of the previous studies in humans utilized the short read 16S sequencing which limits the classification of bacteria mostly to the genus level. Based on our study, the full-length 16S sequencing is recommended for the further analysis of microbiota associated with TB in humans which may help improve the bacterial identification. The *L. johnsonii* was proposed as a potential probiotic showing the anti-inflammatory effect and inhibition of gut pathogens^40^. The mechanism of increasing *L. johnsonii* and the relationship with TB were still unclear. For *E. coprostanoligenes,* it was characterized as a cholesterol-reducing anaerobe^41^. The previous studies reported that low serum cholesterol was associated with TB risk^42^ and TB severity^43^. The possible mechanism was that macrophages that lacked cholesterol possibly impaired the phagocytosis of *Mtbc,* as reported previously^44^. In addition, *B. viscericola* was increased in the TB (+) latent group but not in the active group. The *Barnesiella* in the gut previously showed the potential impact of inhibiting vancomycin-resistant *Enterococcus* in the murine model^45^*.* Previously, *B. viscericola* was firstly isolated from chicken caecum ^46^. In human, the recent study reported that *B. viscericola* was associated with depressed patient caused by polycystic ovary syndrome (PCOS)^47^ but their function was still unclear. Another study suggested that *B. viscericola* is arabinoxylan and pectin degraders which exhibit metabolic interactions with other gut microbes reduced in patient with Crohn’s Disease^48^. These suggest that *B. viscericola* might be the key species involving cross-feeding other beneficial species potentially providing the protective role against the disease progression to active TB since they had a high abundance in the latent group. However, further experiment is needed to confirm their functions. The functional inference analysis of gut microbiota showed few pathways enriched in TB (+) macaques. However, these pathways in gut microbiota seem to have no potential role related to TB.

The pharyngeal microbiota was also changed in TB (+) macaques which the significant bacteria were such as *Haemophilus*, *Prevotella* spp., *Anaerocella delicata*, *Campylobacter gracilis*, and *Treponema putidum*. Some of these bacteria, such as *Prevotella* spp. in human lung have also been linked to TB, while others are less studied. There was limited study focusing on the pharyngeal microbiota in TB patients. Only one study suggested that The Moraxellaceae and Pseudomonadales increased, whereas Bacillales and Lachnospiraceae decreased in nasopharyngeal microbiota of active TB patients^49^. The discrepancies may be due to the different methodologies such as the sample collection method and sequencing approaches. In the current study, the *Haemophilus* was enriched in the TB naive group. Notably, *Haemophilus haemolyticus* was decreased in the TB (+) active group. The decrease in *Haemophilus* was suggested to downregulate the Th1 response in *Mtbc*-infected patients and cause more severity^50^. In contrast, the *Prevotella* species, including *P. buccae*, *P. enoeca*, and *P. heparinolytica,* were consistently enriched in both latent and active groups. Previously, it was found that increased *Prevotella* spp*.* could enhance the production of SCFAs, which can promote FoxP3-expressing regulation, causing the inhibition of protective cytokines (e.g., IFN-γ and IL-17A) production^11^. The SCFA such as butyrate produced by respiratory microbes may also promote IL-10 production and inhibit the *Mtbc*-induced proinflammatory response^51^. This was supported by the functional inference of pharyngeal microbiota presenting that the fermentation pathways generating acetate and butanoate were enriched in the TB (+) active group. Moreover, *Anaerocella delicata* and *Campylobacter gracilis* also increased in both TB (+) active and latent groups. It was reported that *C. gracilis* was related to periodontal diseases and pleuropulmonary infections in humans^52^. The CMP-legionaminate biosynthesis pathway related to *Campylobacter*^53^ was enriched in the TB (+) macaques. The previous study suggested that the metabolites from this pathway could facilitate these pathogens to evade the host immune response^53^. In the TB (+) active group, a potential opportunistic pathogen, *Treponema putidum* was enriched. The *T. putidum* was previously isolated from humans with periodontitis and acute necrotizing ulcerative gingivitis^54^. Therefore, during TB progression, it might cause an increase in opportunistic pathogens. The *Porphyromonas catonae* was decreased in the TB (+) latent and active groups. It was still unclear about the role of this bacterium. This species may be one of the important bacteria in the core pharyngeal microbiota of macaques since it had high abundance and was significantly correlated with other high abundance species, such as *Haemophillus parahaemolyticus*, *P. nanceiensis* and *Neisseria meningtidis* as shown in Fig. 4b. Interestingly, based on the functional inference, the pyruvate fermentation to acetone pathway (PWY-6588) was enriched in TB (+) latent and active groups. It was possible that this pathway may associate with glycolysis pathway in bacteria including *Mtb*c which utilize pyruvate as a substrate for acetyl-CoA production to be the energy source^55^. In this study, the pharyngeal microbiome tends to correlate more with the TB status compared to the gut microbiota since the pharyngeal microbiome showed more significant bacteria and had significant alpha/beta diversities. It is possible that the pharyngeal is a part of the respiratory system which is more related to the infection site compared to the gut.

In summary, this study provided the first report on the association between gut-pharyngeal microbiome and TB stages in a cynomolgus macaque model. The findings showed the difference in microbiota among macaques with different TB stages, implying that the microbiota might be involved with TB pathogenesis and severity. However, the direction of host-microbiota interaction was still unclear. This complex relationship may be a bidirectional interaction. The TB status could affect the microbiome, as suggested in mice model^56^. On the other hand, the TB progression may also cause microbiota change, leading to promoting the positive feedback to dysregulate the immune system, causing more severe disease^57–59^. Based on current analysis, the microbes, particularly in pharyngeal microbiota, might have respiratory immune modulation functions, affecting TB pathogenesis. However, a study focusing on the mechanism should be performed in the future.

## Methods

### Animal experiment statement

All procedures were carried out under the relevant guidelines and regulations. This procedure related to macaques was performed and reported following the ARRIVE guidelines (https://arriveguidelines.org/arrive-guidelines). The animal use protocol related to macaques was reviewed and approved by the Committee of Animal Care and Use at NPRCT-CU (Protocol Review No. 2075007) and the Department of National Parks, Wildlife and Plant Conservation, Thailand (Animal Ethics No. 012/2564, approved by Mahidol Committee, Mahidol University Protocol review No. 009/2564).

### The study cohort

A total of 71 cynomolgus macaques reared at Krabok-Koo Wildlife Breeding Center, Chachoengsao, Thailand (GPS: 13° 28' N, 101° 35' E) were recruited into the present study. These macaques were housed in the same gang cage or in the vicinity of those *Mtbc* infected macaques reported previously (Warit et al., 2020). The macaques were classified into 3 groups: the TB (−) control (n = 45), TB (+) latent (n = 14), and TB (+) active (n = 12) based on the criteria described in Supplementary Table 1. Briefly, the active groups must be positive for Xpert Ultra assay (Cepheid, Sunnyvale, CA, USA), the gold standard of *Mtb*c rapid detection recommended by the World Health Organization (WHO). The latent group must be positive for at least two tests: Tuberculin skin test (TST), Interferon-gamma release assay (IGRA), and Ab-ELISA, but negative for *Mtb*c culture and Xpert Ultra assay. The macaques must be negative for all tests for the TB (−) control group representing TB naive macaque. The characteristics of macaques and *Mtb*c detection results are summarized in Table 1. Among the three groups of macaques, there were no differences in sex, age, and body weight.

**Table 1:** Subject characteristics of macaques with different TB stages based on four *Mtbc* detection methods.

Groups	Sex^a^		Age (year)^b^	BW (kg)^b^	*Mtb*c detections (number of positive/number of tested samples)				
	M	F			Xpert	*Mtb*c culture	TST	IGRA	Ab-ELISA
TB (−) control (n = 45)	38	7	9.0 ± 3.2	5.3 ± 1.8	0/45	0/45	0/45	0/45	0/45
TB (+) latent (n = 14)	10	4	9.5 ± 3.6	5.7 ± 2.2	0/14	0/14	9/14	13/14	6/14
TB (+) active (n = 12)	11	1	9.0 ± 3.2	5.0 ± 1.5	12/12	8/12	3/12	7/12	2/12
*P* value	0.36		0.12	0.67	–	–	–	–	–

^a^The proportions of sex among groups were statistically compared based on the Chi-square test (*P* < 0.05).

^b^The comparisons of age and body weight were carried out based on the Kruskal–Wallis test (*P* < 0.05).

### Specimen collection

For gut microbiota analysis, the fecal swabs were collected, inactivated, and preserved in 1X DNA/RNA Shield solution (Zymo Research, USA) before it was transferred to the laboratory. In addition, the pharyngeal wash (PW) specimen representing the partial-upper respiratory microbiota was collected on the same day with fecal swabs using Phosphate-buffered saline (PBS) to wash the pharynx cavity and aliquot into 1, 1, and 3 ml for performing GenXpert, preserved in 2X DNA/RNA Shield solution for study microbiota and isolation of MTB by cultivation respectively. All specimens were stored at − 80 °C until used for DNA detection, DNA extraction and culture. Study design and animal specimen collection were carried out based on the 3Rs (replacement, reduction and refinement) principle.

### Tuberculin skin test (TST)

For TST, 0.1 ml of the mammalian old tuberculin antigens (*M. bovis* strain 10 and *M. tuberculosis* Aoyama B-strain human tuberculin from Kaketsuken, Japan) diluted with 0.9% sterile normal saline (20,000 IU/0.8 ml) was intradermally injected to upper-eyelid. The TST reaction was evaluated at 24 h, 48 h, and 72 h based on the scoring system (score 0–5) described in Bushmitz et al*.* ^60^. Scorings 1 and 2 were interpreted as negative, while 3, 4 and 5 were positive for TB.

### IGRA determination and interpretation

Four ml of the whole blood was collected from each monkey into a heparinized blood tube. Within 16 h after collection, blood was aliquot (1 ml each) to four tubes; NIL, TB1, TB2 and mitogen (MIT) of QuantiFERON-TB Gold-Plus (QFT-Plus; Catalog no. 622536, QIAGEN, USA). For QFT-Plus assay, NIL tube (negative control) contains no antigen which represents the background IFN-γ. Mitogen tube (positive control) contains mitogen which can induce IFN-γ production. TB1 and TB2 tubes contain *Mtb*c specific antigens which can induce the production of IFN-γ from T cells. TB1 antigen can only detect CD4 T cell response while TB2 antigen can detect CD4 and CD8 T cell response. After mixing and incubation at 37 °C for 20 h, plasma was collected and later used for determination of interferon-gamma (IFN-γ) by using a commercial monkey IFN-γ ELISA pro kit (catalog no. M4210M-1HP-10, Mabtech AB, Sweden). Two kits were combined for this test and named the mIGRA test (Warit et al., 2020). Two criteria of QuantiFERON®-TB Gold Plus (QFT®-Plus) ELISA Package Insert 04/2019 were used to interpret TB detection. Firstly, the IFN-γ level of the MIT stimulated tube must be higher than 10 pg/ml; if it is lower, the test should be interpreted as indeterminate (ID). Secondly, the IFN-γ level of the NIL tube must be lower than those of the TB1 and TB2 tubes; if higher, the interpretation would be ID. For IGRA interpretation, we used a calculation (MDD equation) followed by Parsons et al. (2009) to determine the minimal detectable dose (MDD) value. It was calculated as twice the average IFN-γ level of the NIL tubes. If TB1—NIL or TB2—NIL of each test were greater than the MDD value, it confirmed the TB positive. However, if MIT was lower than NIL, it was interpreted as ID^23^. At the same time, different values of OD at 450 nm between NIL and TB tubes (TB1 and TB2) were also used to determine TB infection. If the OD_450_ of TB was higher than the OD_450_ of NIL (≥ 0.05), it was interpreted as positive. However, if the OD_450_ of MIT was < 0.2 or the OD_450_ of NIL-TB was > 0.2, it was interpreted as ID.

### Antibody-enzyme-linked immunosorbent assay (Ab-ELISA) test

With this test, all sera samples (dilution 1: 200) were used to determine antibody levels against bovine tuberculin PPD (bPPD 3000, Prionics, Netherlands) and human PPD (hPPD, Thai Red Cross, Bangkok) by conventional enzyme-linked immunoassay (ELISA). This method was modified from that recommended by the manufacturer’s protocols. This began by coating the microplate (U-96 maxisorp nunc-immuno plate, cat# 449824, ThermoScientific, Denmark) with 50 µl of 5 µg/ml protein antigen (250 ng) in 0.05 mM carbonate buffer, pH 9.6. The plate was incubated at 4 oC for more than 20 h (h) or overnight. The next day, the plate was washed 3 times by adding 100 µl 0.9% NaCl, and 0.05% Tween20 to each well before blocking with 100 µl 5% skim milk in phosphate buffer saline (PBS) by incubation at 37 oC for 1 h. After washing 3 times with 100 µl 0.9% NaCl and 0.05% Tween20, the plate was filled in 100 µl of 1:200 diluted serum and incubated at 4 oC overnight. The serum was diluted with 1% skim milk and 0.05% Tween20 in PBS. Later, the plate was washed with 100 µl 0.9% NaCl and 0.05% Tween20 for 3 rounds, and the plate was added with 100 µl of 1:5000-diluted goat anti-monkey IgG-HRP conjugated (Abcam cat#112767) in 1% skim milk, PBS, 0.05% Tween20 and incubated at 37 oC for at least 1 h. To develop the color for reading, the plate was washed 3 times with 100 µl of 0.9% NaCl and 0.05% Tween20, added with 50 µl Tetramethyl benzidine (TMB) substrate (Calbiochem# CL07, Millipore, CA, USA) and stood at room temperature for 15 min in a dark place. To stop the color development, 50 µl of 1N sulfuric acid was added. Finally, the indirect ELISA reaction was read by measurement at wavelength 450 nm within 2 h after development. The final reading value was calculated by subtracting the background solution (no sera).

### Xpert® MTB/RIF Ultra

The Xpert® MTB/RIF Ultra assay was performed according to the manufacturer’s protocol. Briefly, 1 ml of pharyngeal wash sample was diluted using the diluent provided by the kit and incubated at room temperature for 15 min. Then, the samples were loaded into the cartridge and analyzed by an automated Xpert machine. This assay was based on semi-nested real-time PCR using primer IS*6110*/IS*1081*, specific to *Mtbc*. The Xpert® software was used to interpret and report the results.

### *Mtb*c culture based on pharyngeal wash samples

After collection, pharyngeal wash was decontaminated and inoculated on LJ media for *Mtbc* cultivation at 37 °C for 1–6 weeks^61^. A positive culture was reported if cream cauliflower-like colonies were identified and positive to one-tube multiplex PCR method^62^. The negative culture was reported when no growth was detected after 2 months of inoculation. All cultural procedures were handled in a biosafety laboratory level 3 (BSL3) containment at the National Institute of Animal Health (NIAH), Bangkok, Thailand.

### The full-length 16S sequencing based on Oxford Nanopore Technologies

Total DNA was extracted using a ZymoBIOMICS DNA Miniprep Kit (Zymo Research, USA) per the manufacturer's protocol. The bacteria full-length 16S rDNA (V1-V9 regions) was amplified using the primers modified from a previous publication^63^, which comprised of the 3' specific target sequences (underlined) and 5' nanopore adaptors as follows: 16S_27F 5'-TTTCTGTTGGTGCTGATATTGCAGRGTTYGATYMTGGCTCAG-3' and 16S_1492R 5'-ACTTGCCTGTCGCTCTATCTTCCGGYTACCTTGTTACGACTT-3'. The PCR reaction contained 0.25 µM of each forward and reverse primer, 0.2 µM of dNTPs, 0.4 U of Phusion™ Plus DNA Polymerase (Thermo Fisher Scientific, USA) and 10 ng of genomic DNA. The thermal condition for PCR was 98 °C for 30 s, 25 cycles of 98 °C for 10 s, 60 °C for 10 s, 72 °C for 45 s, and a final extension at 72 °C for 5 min. The PCR products were subsequently amplified by 5-cycle barcode PCR based on PCR Barcoding Expansion 1–96 (EXP-PBC096) kit (Oxford Nanopore Technologies, UK). The PCR profile of the barcoding step was 98 °C for 30 s, 5 cycles of 98 °C for 10 s, 60 °C for 10 s, 72 °C for 60 s, and a final extension at 72 °C for 5 min. The DNA library was then purified using the QIAquick PCR Purification Kit (Qiagen, Germany) and measured for quantity by Quant-iT™ dsDNA HS Assay Kits (Invitrogen, USA). The DNA was equimolarly pooled and size-selected using 0.5 × Agencourt AMPure XP beads (Beckman Coulter, USA). One µg of the barcoded library was ligated with Nanopore adaptors using the Ligation Sequencing Kit (SQK-LSK112, Oxford Nanopore Technologies, UK) and sequenced on the R10.4 (Q20 +) flow cell using the MinION Mk1C sequencer (Oxford Nanopore Technologies, UK). The sequencing reads summary is described in Supplementary Table 2.

### Bioinformatic analysis of full-length 16S sequencing of gut and pharyngeal microbiota

The FAST5 files from Nanopore sequencing was basecalled using guppy basecaller v6.1.2 (Oxford Nanopore Technologies, UK) with super-accuracy (SUP) mode. The quality of the FASTQ sequence was primarily examined by MinIONQC^64^. Then, the FASTQ reads were demultiplexed and adaptor trimmed using Porechop v0.2.4 (https://github.com/rrwick/Porechop). The filtered reads of each sample were then used for read clustering, polishing, and taxonomic identification based on NanoCLUST^65^ pipeline. The taxonomic classification was performed based on the Blastn algorithm using the reference sequences retrieved from the RDP database (version 11.5). The outputs of bacterial abundance were then subsequently analyzed by QIIME2 v2021.2^66^ to generate a collapsed taxa table. The Microbiome Analyst^67^ was also used to analyse the alpha and beta diversity of microbiota. The differential abundance analysis was performed by LEfSe^68^ method (LDA score > 2 and *P* < 0.05) based on the galaxy web server (https://huttenhower.sph.harvard.edu/galaxy/). The cooccurrence network analysis of bacteria within gut and pharyngeal microbiota was carried out by SparCC method (r < 0.3, *P* < 0.05)^69^ using the plugin in Microbiome Analyst. The polished representative sequence of clusters derived from NanoCLUST was used for functional inference analysis using PICRUSt2^70^. The abundance of predicted pathways based on MetaCyc annotation^71^ was statistically tested by independent *t*-test to compare the pathway abundance between control groups against active and latent groups.

### Supplementary Information

Supplementary Information.

## Data Availability

The sequencing datasets from the current study are publicly available in the NCBI Sequence Read Archive (SRA), BioProject ID: PRJNA1030943.
