# Supplementary material for: Full-length 16S rDNA sequencing based on Oxford Nanopore Technologies revealed the association between gut-pharyngeal microbiota and tuberculosis in cynomolgus macaques
Source: Sci Rep. 2024 Feb 10;14:3404. doi: 10.1038/s41598-024-53880-w (PMC10858278; doi:10.1038/s41598-024-53880-w)
Supplement: Supplementary file 1 — Supplementary Information. [file 41598_2024_53880_MOESM1_ESM.pdf]

Full-length 16S rDNA sequencing based on Oxford Nanopore Technologies revealed the association between gut-pharyngeal microbiota and tuberculosis in cynomolgus macaques

Vorthon Sawaswong[1,2], Prangwalai Chanchaem[1], Pavit Klomkliew[1], Suwatchareeporn Rotcheewaphan[1,3], Suthirote Meesawat[4,5], Taratorn Kemthong[4,5], Mutchamon Kaewparuehaschai[6], Kirana Noradechanon[6], Monya Ekatat[7], Reka Kanitpun[7], Prapaporn Srilohasin[8,9], Saradee Warit[10], Angkana Chaiprasert[8], Suchinda Malaivijitnond[4,5], Sunchai Payungporn[1,*]

[1] Center of Excellence in Systems Microbiology, Department of Biochemistry, Faculty of Medicine, Chulalongkorn University, Bangkok, 10330, Thailand.

[2] Department of Biochemistry, Faculty of Science, Mahidol University, Bangkok 10400, Thailand.

[3] Department of Microbiology, Faculty of Medicine, Chulalongkorn University, Bangkok, 10330, Thailand.

[4] National Primate Research Center of Thailand, Chulalongkorn University, Saraburi, 18110, Thailand.

[5] Department of Biology, Faculty of Science, Chulalongkorn University, Bangkok, 10330, Thailand.

[6] Wildlife Conservation Office, Department of National Parks Wildlife and Plant Conservation, Bangkok, 10900, Thailand.

[7] National Institute of Animal Health (NIAH), Bangkok, 10900, Thailand.

[8] Office for Research, Faculty of Medicine Siriraj Hospital, Mahidol University, Bangkok, 10700, Thailand.

[9] Department of Microbiology, Faculty of Medicine Siriraj Hospital, Mahidol University, Bangkok, 10700, Thailand.

[10] Industrial Tuberculosis Team, Industrial Medical Molecular Biotechnology Research Group, National Center for Genetic Engineering and Biotechnology, National Science and Technology Development Agency, Pathum Thani, 12120, Thailand.

* Corresponding author:
Associate Professor Sunchai Payungporn, Ph.D.
Center of Excellence in Systems Microbiology,
Department of Biochemistry, Faculty of Medicine, Chulalongkorn University
1873 Rama IV Road, Patumwan, Bangkok 10330, Thailand
E-mail: sp.medbiochemcu@gmail.com Tel: +662-256-4482

Supplementary Materials

Supplementary Table 1 The grouping criteria of macaques based on the stages of TB

<table>
<tr><th rowspan="2">Diagnostic method</th><th colspan="3">Groups</th></tr>
<tr><th>TB (-) control</th><th>TB (+) latent</th><th>TB (+) active</th></tr>
<tr><td>Xpert Ultra</td><td>-</td><td>-</td><td>+</td></tr>
<tr><td>Mtbc culture</td><td>-</td><td>-</td><td>+/-</td></tr>
<tr><td>Tuberculin skin test (TST)</td><td>-</td><td rowspan="3">+ at least 2 tests</td><td>+/-</td></tr>
<tr><td>Interferon-gamma release assay (IGRA)</td><td>-</td><td>+/-</td></tr>
<tr><td>Ab-ELISA</td><td>-</td><td>+/-</td></tr>
</table>

Supplementary Table 2 The raw and classified reads summary from full-length 16S sequencing

Source	Groups	Raw reads	Filtered and Classified reads	% Read classified
Pharynx	control	8,713 ± 3,276	4,695 ± 1,953	54.5 ± 13.2
	latent	14,203 ± 7,358	7,955 ± 5,830	52.8 ± 13.7
	active	12,035 ± 4,630	7,967 ± 3,829	66.6 ± 14.9
Feces	control	6,049 ± 2,803	4,695 ± 1,953	80.6 ± 10.4
	latent	10,092 ± 7,440	7,955 ± 5,830	79.3 ± 7.7
	active	11,387 ± 6,601	7,967 ± 3,829	74.1 ± 11.1

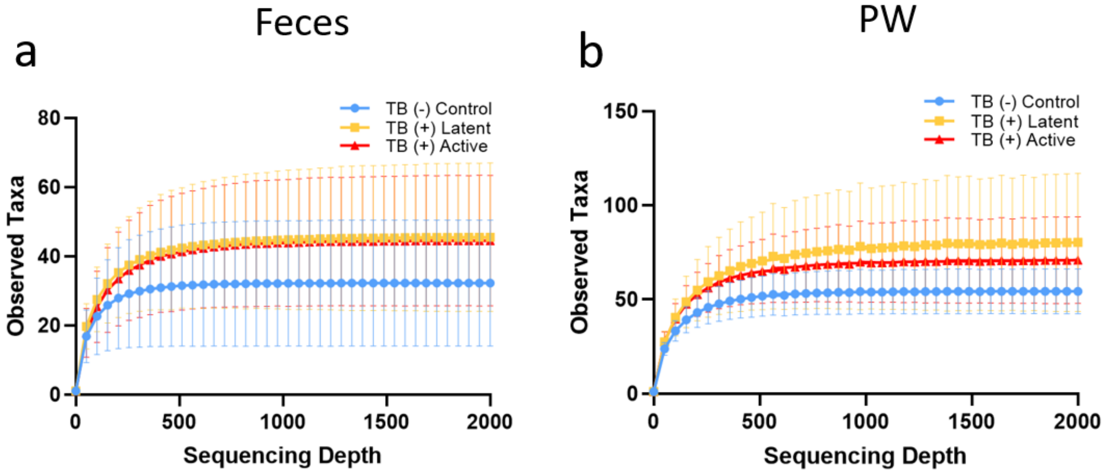

Supplementary Figure S1 Rarefaction curve of gut and pharyngeal microbiota classified based on 16S full-length sequencing using Oxford Nanopore Technologies. The result of rarefaction analysis was represented by a line graph plotting the number of observed taxa against the sequencing depth in (a) gut and (b) pharyngeal microbiota.

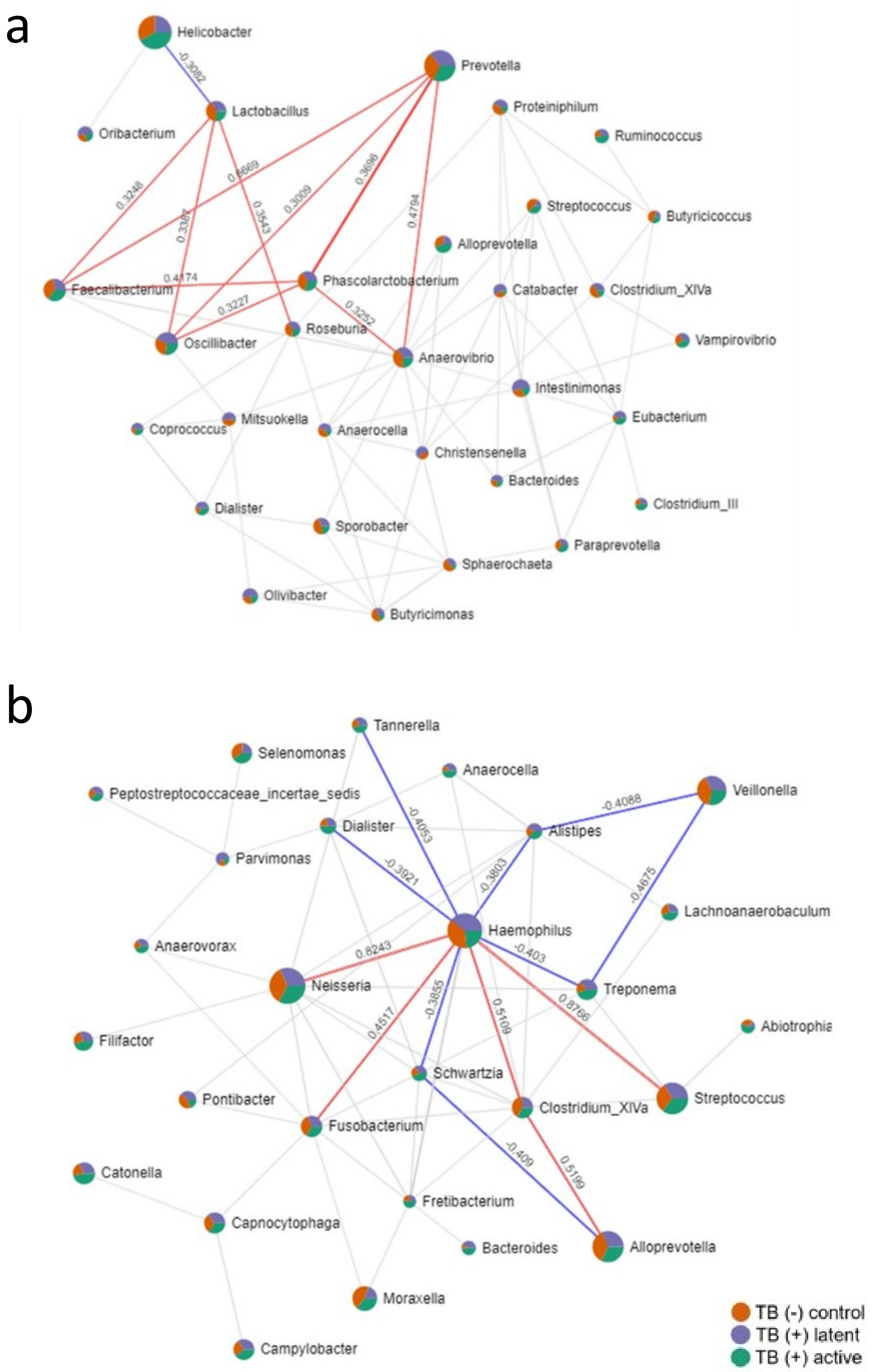

Supplementary Figure S2 Correlation network analysis of gut and pharyngeal microbiota in long-tailed macaques at genus level. The correlation network constructed based on the SPARCC method represents the relationship of bacterial species in the gut microbiota of macaques (*r*>0.3, <0.05). The colors of the edges represent correlation types: positive (red) and negative (blue) correlation. The nodes were designated as pie charts indicating the relative abundance of each bacterium in macaques with different TB stages: negative control (orange), latent (purple) and active (green).

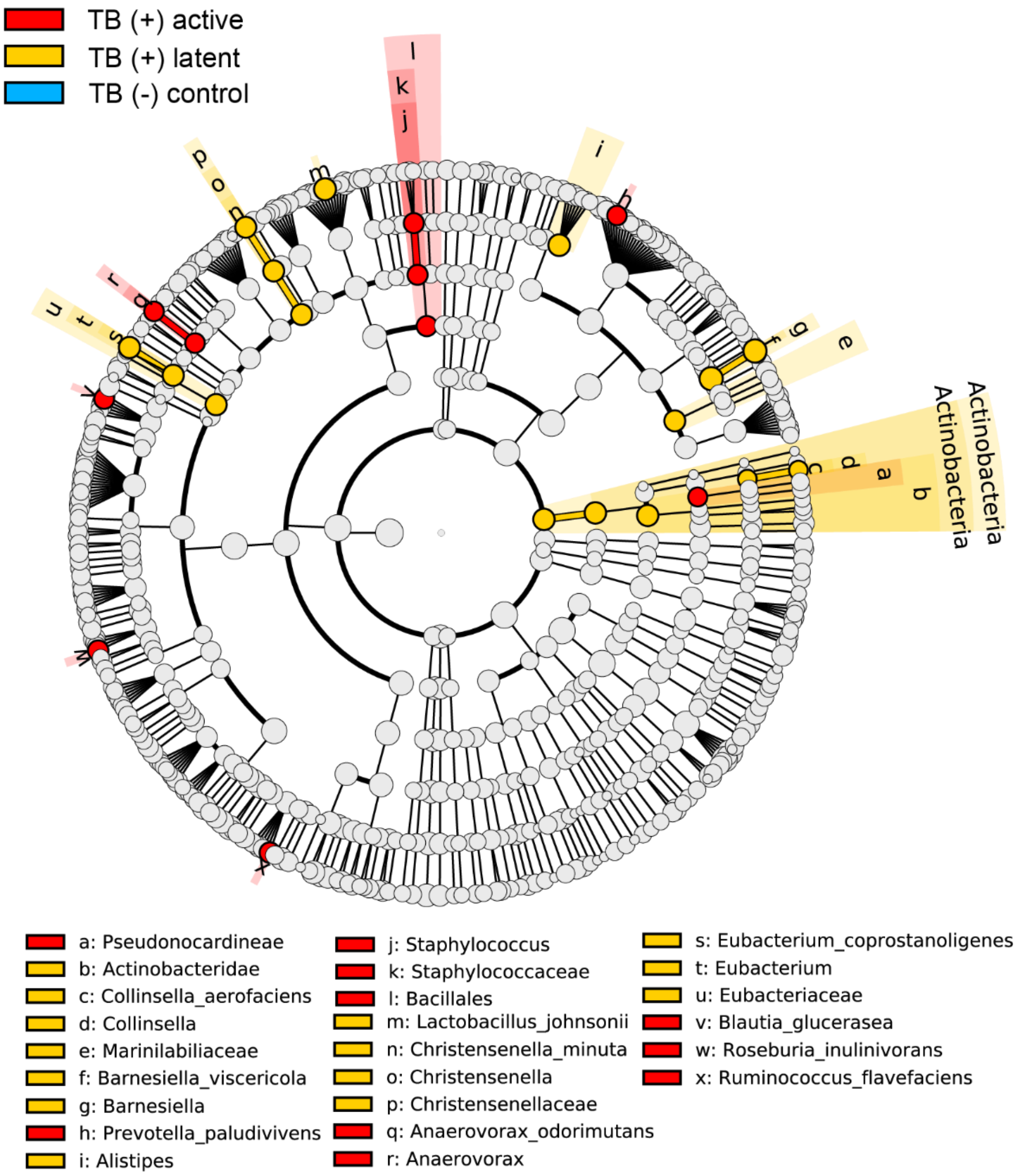

Supplementary Figure S3 Differential abundance analysis comparing bacterial taxa in the gut of macaques with different TB stages. The cladogram showed the results from LEfSe analysis showing the significantly differential taxa (LDA score >2, *P*<0.05) in the gut of macaques with different TB stages.

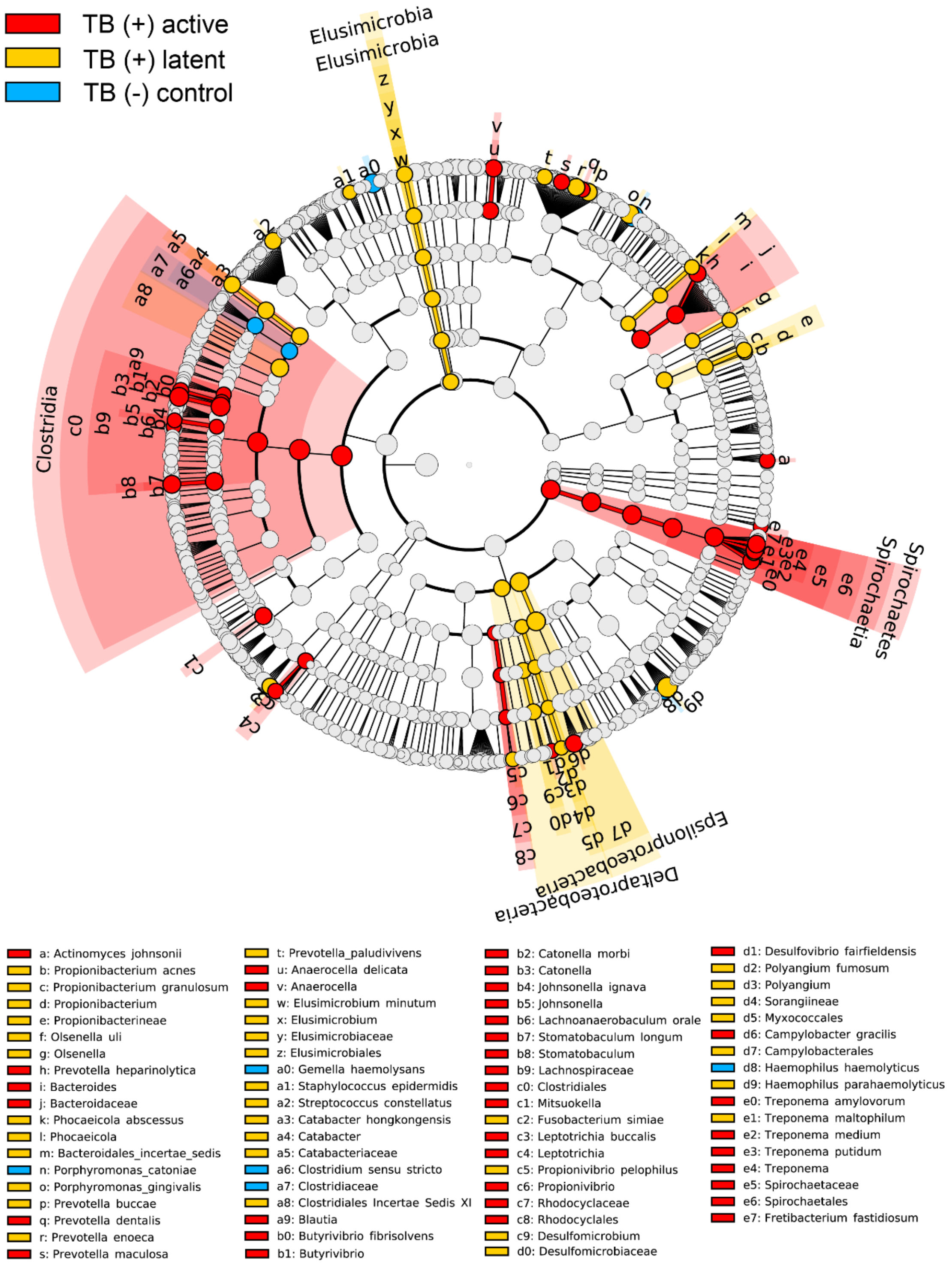

Supplementary Figure S4 Differential abundance analysis comparing bacterial taxa in the pharynx of macaques with different TB stages. The cladogram showed the results from LEfSe analysis showing the significantly differential taxa (LDA score >2, *P*<0.05) in the pharyngeal microbiota of macaques with different TB stages.

Supplementary Table 3 The results of multivariate analysis based on linear model (LM) using the gender as the covariate to control the effect of gender on the relationship between gut microbiome profile and TB stages.

Gut bacteria	Control vs Latent		Control vs Active	
	Log_2FC	*P* value	Log_2FC	*P* value
Eubacterium_coprostanoligenes	-1.110	0.011 *	-1.310	0.004 *
Ruminococcus_flavefaciens	0.168	0.686	-1.060	0.018 *
Prevotella_ruminicola	0.794	0.108	1.200	0.023 *
Alloprevotella_rava	0.176	0.722	-0.890	0.092
Clostridium_thermocellum	-0.537	0.219	-0.701	0.129
Sarcina_ventriculi	-1.120	0.001 *	-0.508	0.133
Dialister_succinatiphilus	-0.937	0.011 *	-0.541	0.158
Coprococcus_eutactus	-0.792	0.034 *	-0.541	0.164
Sphaerochaeta_globosa	-0.031	0.949	0.678	0.192
Ruminococcus_bromii	-0.799	0.087	-0.631	0.197
Barnesiella_viscericola	-1.960	0.000 *	-0.701	0.212

* Asterisks indicate the significant taxa ($P<0.05$); Bold text indicates the significant taxa from standard analysis (Figure 5)

Supplementary Table 4 The results of multivariate analysis based on linear model (LM) using the gender as the covariate to control the effect of gender on the relationship between pharyngeal microbiome profile and TB stages.

Pharyngeal bacteria	Control vs Latent		Control vs Active	
	Log_2FC	*P* value	Log_2FC	*P* value
Prevotella_heparinolytica	-1.270	0.002 *	-1.510	0.001 *
Treponema_medium	-0.745	0.146	-1.740	0.002 *
Treponema_putidum	-1.020	0.018 *	-1.420	0.002 *
Streptococcus_sinensis	-0.187	0.602	-1.150	0.003 *
Catonella_morbi	-0.645	0.098	-1.220	0.004 *
Campylobacter_gracilis	-1.120	0.014 *	-1.340	0.005 *
Porphyromonas_catoniae	0.548	0.024 *	0.715	0.006 *
Haemophilus_parahaemolyticus	0.113	0.723	0.931	0.007 *
Dialister_invisus	-0.276	0.486	-1.110	0.009 *
Haemophilus_haemolyticus	0.486	0.395	1.530	0.013 *
Anaerocella_delicata	-0.957	0.038 *	-1.180	0.016 *
Fretibacterium_feline	-0.214	0.537	-0.891	0.017 *
Prevotella_oris	-0.410	0.298	-1.000	0.018 *
Gemella_haemolysans	0.106	0.590	0.497	0.019 *
Prevotella_fusca	0.022	0.964	-1.190	0.021 *
Filifactor_alocis	-0.204	0.671	-1.180	0.022 *
Prevotella_enoeca	-1.020	0.010 *	-0.944	0.023 *
Prevotella_buccae	-1.210	0.006 *	-1.020	0.027 *
Peptostreptococcus_stomatis	0.052	0.890	-0.898	0.027 *
Tannerella_forsythia	-0.560	0.135	-0.836	0.036 *
Oribacterium_sinus	-0.186	0.751	-1.270	0.043 *
Campylobacter_showae	-0.545	0.280	-1.090	0.044 *
Moraxella_nonliquefaciens	0.747	0.111	0.981	0.048 *
Porphyromonas_gingivalis	-1.480	0.014 *	-1.010	0.108
Streptococcus_cristatus	-1.510	0.003 *	-0.739	0.158
Actinobacillus_pleuropneumoniae	-1.250	0.004 *	-0.623	0.164
Treponema_vincentii	-0.813	0.038 *	-0.244	0.548
Parvimonas_micra	-1.020	0.047 *	0.053	0.921

* Asterisks indicate the significant taxa ($P<0.05$); Bold text indicates the significant taxa from standard analysis (Figure 5)

Supplementary Table 5 The significant taxa from inferred absolute abundance analysis of gut microbiome carried out by ANCOM-BC

Gut bacteria	Latent vs Control		Active vs Control	
	Log_2FC	*P* value	Log_2FC	*P* value
Prevotella saccharolytica	-0.173	0.752	-1.154	0.000 *
Alloprevotella rava	0.300	0.632	1.717	0.000 *
Eubacterium coprostanoligenes	1.573	0.005 *	2.080	0.001 *
Christensenella minuta	0.986	0.097	-0.833	0.001 *
Pseudoflavonifractor capillosus	0.806	0.164	-0.743	0.001 *
Megasphaera elsdenii	0.778	0.162	1.973	0.002 *
Ruminococcus flavefaciens	0.140	0.719	1.653	0.003 *
Helicobacter macacae	-1.428	0.147	2.089	0.003 *
Paraprevotella clara	0.630	0.253	1.568	0.006 *
Eubacterium hallii	-0.588	0.006 *	-0.570	0.007 *
Ruminobacter amylophilus	0.440	0.401	-0.675	0.008 *
Ruminococcus torques	0.342	0.330	1.382	0.009 *
Lactobacillus johnsonii	2.512	0.000 *	1.704	0.009 *
Anaerovorax odorimutans	0.303	0.357	1.292	0.012 *
Blautia glucerasea	-0.069	0.541	1.235	0.015 *
Clostridium celerecrescens	-0.236	0.426	-0.507	0.019 *
Lactobacillus murinus	-0.138	0.685	-0.425	0.019 *
Roseburia inulinivorans	0.740	0.160	1.298	0.024 *
Luteolibacter algae	0.529	0.291	-0.439	0.027 *
Barnesiella viscericola	2.429	0.000*	1.265	0.040 *
Intestinimonas butyriciproducens	1.618	0.021 *	1.275	0.040 *
Prevotella ruminicola	-0.720	0.214	-1.031	0.049 *
Treponema berlinense	-0.850	0.000 *	-0.269	0.535
Lactobacillus salivarius	-0.807	0.002 *	-0.443	0.260
Oribacterium sinus	1.650	0.003 *	0.038	0.957
Sarcina ventriculi	1.703	0.004 *	1.009	0.094
Acetobacteroides hydrogenigenes	-0.550	0.010 *	-0.183	0.641
Coprococcus eutactus	1.380	0.012 *	1.092	0.065
Dialister succinatiphilus	1.570	0.012 *	0.921	0.190
Streptococcus lutetiensis	-1.320	0.017 *	-0.635	0.358
Eubacterium ruminantium	1.162	0.018 *	0.507	0.218
Campylobacter troglodytis	-0.597	0.018 *	0.311	0.644
Butyrivibrio fibrisolvens	1.265	0.019 *	0.465	0.350
Ruminococcus bromii	1.394	0.020 *	1.047	0.091
Clostridium thermocellum	1.146	0.024 *	1.061	0.084
Clostridium nexile	-0.460	0.026 *	0.116	0.774
Ruminococcus gnavus	-0.457	0.030 *	0.294	0.579
Blautia obeum	1.021	0.033 *	0.239	0.619
Alkaliflexus imshenetskii	0.779	0.040 *	0.482	0.212
Lactobacillus gallinarum	1.102	0.042 *	0.143	0.764
Prevotella fusca	1.245	0.048 *	0.717	0.250

* Asterisks indicate the significant taxa ($P<0.05$); Bold text indicates the significant taxa from standard analysis (Figure 5)

Supplementary Table 6 The significant taxa from inferred absolute abundance analysis of pharyngeal microbiome carried out by ANCOM-BC

Pharyngeal bacteria	Latent vs Control		Active vs Control	
	Log_2FC	*P* value	Log_2FC	*P* value
Filifactor alocis	0.360	0.576	1.658	0.000 *
Catonella morbi	1.017	0.015 *	1.335	0.000 *
Treponema putidum	1.428	0.020 *	2.093	0.000 *
Prevotella enoeca	1.867	0.002 *	1.765	0.000 *
Campylobacter gracilis	1.686	0.006 *	1.998	0.000 *
Treponema amylovorum	1.548	0.001 *	1.858	0.001 *
Dialister invisus	0.776	0.195	1.879	0.001 *
Prevotella heparinolytica	1.973	0.000 *	1.943	0.001 *
Campylobacter showae	0.901	0.125	1.683	0.001 *
Prevotella buccae	1.609	0.010 *	1.795	0.001 *
Prevotella falsenii	0.314	0.387	0.660	0.002 *
Streptococcus sinensis	0.445	0.403	1.739	0.004 *
Fretibacterium feline	0.698	0.172	1.495	0.005 *
Tannerella forsythia	1.203	0.032 *	1.503	0.006 *
Anaerocella delicata	1.389	0.019 *	1.666	0.007 *
Prevotella oris	0.632	0.282	1.488	0.007 *
Prevotella fusca	0.459	0.427	1.553	0.007 *
Moraxella nonliquefaciens	-0.712	0.271	-1.536	0.008 *
Haemophilus influenzae	1.586	0.017 *	1.613	0.009 *
Treponema medium	1.045	0.051	1.568	0.015 *
Natronoflexus pectinivorans	0.318	0.429	-0.423	0.016 *
Johnsonella ignava	0.953	0.003 *	0.879	0.019 *
Moraxella lacunata	0.850	0.141	1.382	0.021 *
Lactobacillus salivarius	-0.514	0.022 *	-0.489	0.021 *
Peptostreptococcus stomatis	0.287	0.554	1.292	0.021 *
Prevotella melaninogenica	-0.055	0.934	-1.286	0.021 *
Porphyromonas gingivalis	1.889	0.001 *	1.364	0.023 *
Stomatobaculum longum	0.264	0.613	1.671	0.027 *
Mitsuokella jalaludinii	0.673	0.194	1.307	0.028 *
Butyrivibrio fibrisolvens	0.754	0.028 *	1.089	0.031 *
Lactobacillus animalis	-0.359	0.304	-0.572	0.031 *
Prevotella baroniae	0.512	0.183	1.100	0.032 *
Actinomyces johnsonii	0.842	0.020 *	0.978	0.034 *
endosymbiont of	-0.135	0.766	-0.492	0.036 *
Meniscus glaucopis	0.111	0.761	-0.363	0.036 *
Haemophilus haemolyticus	0.131	0.838	-1.451	0.037 *
Bacteroides eggerthii	-0.100	0.766	-0.366	0.039 *
Oribacterium sinus	0.784	0.174	1.351	0.040 *
Barnesiella viscericola	0.869	0.058	1.252	0.040 *
Lactobacillus mucosae	-0.400	0.047 *	-0.375	0.044 *
Neisseria oralis	0.625	0.233	-0.876	0.045 *
Schwartzia succinivorans	1.094	0.050 *	1.200	0.048 *
Lachnoanaerobaculum saburreum	0.862	0.117	1.192	0.050 *
Prevotella conceptionensis	1.277	0.000 *	0.546	0.347
Haemophilus segnis	0.903	0.000 *	0.273	0.343
Streptococcus constellatus	2.085	0.000 *	1.095	0.055

Catabacter hongkongensis	1.929	0.000 *	0.721	0.120
Treponema maltophilum	1.546	0.000 *	0.539	0.152
Streptococcus cristatus	2.046	0.002 *	0.909	0.226
Prevotella intermedia	-0.703	0.002 *	-0.304	0.466
Neisseria zoodegmatis	-0.686	0.002 *	-0.344	0.362
Parabacteroides merdae	1.477	0.004 *	0.432	0.365
Olsenella uli	1.043	0.004 *	0.340	0.237
Bacteroides salanitronis	1.199	0.004 *	0.490	0.209
Treponema vincentii	1.651	0.004 *	0.650	0.313
Phocaeicola abscessus	0.955	0.006 *	0.025	0.720
Aggregatibacter actinomycetemcomitans	1.272	0.006 *	0.791	0.082
Propionivibrio pelophilus	0.922	0.008 *	0.532	0.118
Actinobacillus pleuropneumoniae	1.854	0.009 *	0.894	0.182
Clostridium cellobioparum	1.302	0.011 *	0.712	0.203
Helicobacter pametensis	1.436	0.014 *	0.382	0.342
Desulfovibrio fairfieldensis	0.713	0.014 *	0.580	0.109
Fusobacterium canifelinum	1.197	0.015 *	0.681	0.197
Staphylococcus epidermidis	0.776	0.016 *	0.349	0.187
Alistipes finegoldii	1.412	0.017 *	0.977	0.140
Propionibacterium acnes	0.960	0.019 *	0.425	0.206
Prevotella saccharolytica	1.009	0.019 *	0.899	0.101
Propionibacterium granulosum	1.013	0.020 *	0.299	0.169
Prevotella bivia	1.313	0.021 *	0.248	0.663
Anaerovorax odorimutans	1.220	0.026 *	0.463	0.472
Tissierella praeacuta	0.835	0.026 *	0.581	0.244
Elusimicrobium minutum	1.105	0.027 *	-0.281	0.073
Filifactor villosus	1.009	0.029 *	0.446	0.251
Prevotella aurantiaca	1.326	0.029 *	0.624	0.183
Gemella haemolysans	0.345	0.030 *	-0.094	0.576
Selenomonas noxia	1.081	0.030 *	0.890	0.141
Campylobacter concisus	1.051	0.032 *	0.176	0.694
Polyangium fumosum	0.649	0.032 *	-0.046	0.637
Sphaerochaeta globosa	0.770	0.032 *	0.204	0.428
Parvimonas micra	1.331	0.032 *	-0.071	0.893
Fretibacterium fastidiosum	0.728	0.034 *	0.654	0.109
Acinetobacter radioresistens	-0.510	0.037 *	-0.104	0.809
Neisseria elongata	1.159	0.045 *	-0.217	0.650
Fusobacterium simiae	1.030	0.046 *	0.587	0.169
Capnocytophaga leadbetteri	0.491	0.047 *	0.569	0.229
Coraliomargarita akajimensis	0.498	0.048 *	0.025	0.720
Bergeyella zoohelcum	0.940	0.050 *	0.145	0.665

* Asterisks indicate the significant taxa (P<0.05); Bold text indicates the significant taxa from standard analysis (Figure 5)
